# Which Coronary Artery Should Be Preferred for Starting the Coronary Spasm Provocation Test?

**DOI:** 10.3390/life13102072

**Published:** 2023-10-17

**Authors:** Hiroki Teragawa, Yuko Uchimura, Chikage Oshita, Yu Hashimoto, Shuichi Nomura

**Affiliations:** Department of Cardiovascular Medicine, JR Hiroshima Hospital, 3-1-36, Futabanosato, Higashi-ku, Hiroshima 732-0057, Japan; yuuko-uchimura@jrhh.or.jp (Y.U.); chikage-ooshita@jrhh.or.jp (C.O.); yu-hashimoto@jrhh.or.jp (Y.H.); shuichi-nomura@jrhh.or.jp (S.N.)

**Keywords:** coronary spasm, multivessel spasm, spasm provocation test, vasospastic angina

## Abstract

Background: The spasm provocation test (SPT) is a critical test for diagnosing vasospastic angina (VSA). However, the choice of vessel to be preferred for initiating the SPT—the right coronary artery (RCA) or the left coronary artery (LCA)—is unclear. This study aimed to assess SPT results including SPT-related complications while initiating the SPT in the RCA and LCA. Methods: We enrolled 225 patients who underwent coronary angiography and SPTs. The SPT was first performed in the RCA in 133 patients (RCA group) and the LCA in 92 patients (LCA group). We defined VSA as >90% narrowing of the coronary artery during the SPT, accompanied by chest pain and/or ST–T changes on the electrocardiogram. When coronary spasm occurs in two or more major coronary arteries, it is referred to as a multivessel spasm (MVS). SPT-related complications comprised atrial fibrillation, ventricular fibrillation, and unstable hemodynamics following catecholamine use. Analyses using propensity score matching (PSM) were performed in 120 patients. Results: No significant differences in the frequencies of VSA and complications were observed between the two groups (RCA: 79% and 19%, respectively; LCA: 85% and 22%, respectively). In both groups, spasms were most frequently provoked in the left anterior descending coronary artery (both *p* < 0.001) whereas spasms in the left circumflex coronary artery (LCX) were higher in the LCA group than in the RCA group (*p* = 0.015). Furthermore, no significant difference in the frequency of MVS was observed between both groups (RCA: 50%, LCA: 62%; *p* = 0.122). After PSM, no significant difference in the frequencies of VSA and complications were observed between the two groups (RCA: 82% and 15%, respectively; LCA: 88% and 18%, respectively). The frequencies of LCX spasms (RCA: 8%, LCA: 23%; *p* = 0.022) and MVS (RCA: 40%, LCA: 62%; *p* = 0.020) were higher in the LCA group than in the RCA group. Conclusions: Although the diagnostic rate of VSA and frequency of SPT-related complications were similar in the two groups, the frequency of MVS was higher in the LCA group than in the RCA group because of the increase in the number of LCX spasms. A routine SPT may be started from the LCA.

## 1. Introduction

A coronary spasm refers to a transient constriction of the epicardial coronary artery, leading to myocardial ischemia [1,2,3]. Coronary spasm is increasingly being recognized as one of the main causes of ischemia with nonobstructive coronary artery disease or myocardial infarction with nonobstructive coronary arteries (MINOCA) [4]. The diagnosis of vasospastic angina (VSA) is based on the detection of transient ST–T changes on an electrocardiogram (ECG) in addition to chest pain occurring at night or during rest [5,6]. However, transient ST–T changes on an ECG may not be detected even in patients experiencing chest symptoms. For this reason, in clinical settings, the diagnosis of VSA is often made by performing the spasm provocation test (SPT). Furthermore, coronary angiography (CAG) and the SPT can also provide prognostic information in addition to being tools for diagnosing VSA [7,8,9,10].

Although the SPT is a well-established test [5,6,11], a clear consensus regarding some aspects is lacking, such as the dose of the provocative drugs [6,12], whether it should be performed during the acute phase of MINOCA [6,13], and whether the coronary microvascular function test should be performed before or after the SPT [4,6,14]. Although multivessel spasm (MVS) is a significant factor in the activity and prognosis of VSA [8,15], and making the diagnosis of MVS is recommended by the guidelines [6], whether to start with the left coronary artery (LCA) or right coronary artery (RCA) has also not been well established. According to a recent guideline [6], the SPT can be started from either the RCA or LCA; however, because the LCA contains two major coronary arteries, the test is often started from the LCA.

In this study, we compared the frequency of coronary spasms, particularly MVS, and the frequency of SPT-related complications in patients who underwent the SPT for suspected VSA at our institution and in whom the SPT was initiated from the RCA or LCA. Our findings may provide insights that may help inform the choice of whether the SPT should be started from the LCA or RCA.

## 2. Materials and Methods

### 2.1. Study Participants

**Figure 1:**
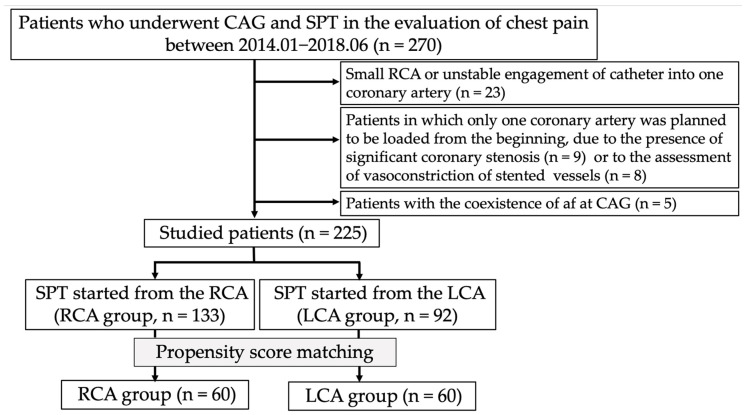
Flowchart of the study protocol. Abbreviations: af, atrial fibrillation; CAG, coronary angiography; LCA, left coronary artery; RCA, right coronary artery; SPT, spasm provocation test.

This was a retrospective observational study. Between January 2014 and June 2018, 270 patients underwent SPT to evaluate chest pain, mainly during rest, at our institution. Up until April 2016, at our institution, spasms were initially provoked in the RCA. The methodology was subsequently changed to begin provocation from the LCA. Patients (*n* = 23) who could not undergo spasm provocation in the RCA because of its small territory or because a catheter could not be inserted into its ostium were excluded from this study. Additionally, we excluded 17 patients in whom only one coronary artery was originally subjected to the SPT because of the presence of vasoconstriction of stented lesions (*n* = 8) or significant coronary stenosis at other coronary artery trees (*n* = 9). We also excluded five patients with persistent or chronic atrial fibrillation (Af). Therefore, we finally enrolled 225 patients in this study (Figure 1). Among them, the SPT was initiated from the RCA in 133 patients (RCA group) and from the LCA in 92 patients (LCA group). All patients provided written informed consent regarding the SPT and CAG. The Ethics Committee of our institution approved the study protocol (2022–39), and consent was confirmed by an opt-out method on the homepage (http://www.jrhh.sakura.ne.jp/annnai/torikumi.html, accesses on 4 January 2023) because of the retrospective nature of this study.

### 2.2. SPT

The steps taken at our institution for the SPT have been previously discussed [15,16,17]. Briefly stated, the SPT was performed after a standard diagnostic CAG. Through the internal jugular vein or medial cubital vein, a 5-French gauge (Fr) temporary pacing catheter (Bipolar Balloon Catheter, B. Braun, Melsungen, Germany) was introduced into the right ventricle and set to 50 beats per minute. Acetylcholine (ACh) was administered into the LCA for 20 s every 3 min throughout the SPT for the LCA. If no coronary spasm was elicited by 100 µg of ACh, a maximum of 200 µg ACh was injected into the LCA. Then, 20 and 50 µg of ACh were injected into the RCA for 20 s at 3 min intervals during the SPT for the RCA. If no coronary spasm was elicited by 50 µg of ACh, a maximum of 80 µg was injected into the RCA. Methylergometrine maleate (EM) (Fuji Pharma, Tokyo, Japan) was injected into the LCA in some patients following the ACh provocations in doses of 20, 40, and 60 µg for the same duration and period and/or into the RCA in doses of 30 and 50 µg for the same duration and period. Furthermore, in the so-called “sequential provocation” described by Sueda et al. [18], another maximal dosage of ACh was administered into the LCA or RCA. CAG was performed immediately after each ACh infusion or whenever chest pain and/or ST–T alterations on ECG appeared. If a coronary spasm was elicited but resolved spontaneously, an SPT for another coronary artery was performed without intracoronary infusion of nitroglycerin (NTG). If provocative drug infusion into one coronary artery resulted in severe coronary spasm or hemodynamic instability, an intracoronary infusion of NTG with a dose of 0.3 mg was administered at the conclusion of the SPT; otherwise, the next SPT was performed in a different coronary artery.

### 2.3. Factors Associated with CAG and Coronary Spasm

The aforementioned methodology was used to measure the diameters of the coronary arteries [16]. The average value was used for the analysis after each measurement was made three times. Atherosclerotic lesions and substantial coronary stenosis were defined as lesions with stenosis of >20% and >50%, respectively. VSA was defined as a coronary artery constriction of >90% on CAG performed during the SPT, together with the presence of usual chest symptoms and/or an ST-segment deviation on ECG [6]. MVS is defined as a coronary spasm affecting two major coronary arteries. When the SPT for a different coronary artery was not provoked after the inevitable use of NTG, the diagnosis of MVS could not be verified, and the outcome was later designated as “not diagnosed (ND)” [15]. A focal spasm is described as a transient vasoconstriction of >90% within the confines of a single isolated coronary segment, based on the classification of the American Heart Association [7]. For the LCA, the ACh doses for spasm provocation were 50 µg (low dose), 100 µg (moderate dose), and 200 µg (high dose). For the RCA, the corresponding doses were 20 µg (low dose), 50 µg (moderate dose), and 80 µg (high dose). The frequency of coronary spasms in the left circumflex coronary artery (LCX), left anterior descending coronary artery (LAD), and RCA and the occurrence of severe complications, such as prolonged hemodynamic instability requiring intravenous catecholamines, ventricular fibrillation (Vf), pulseless ventricular tachycardia (pVT), cardiac arrest, and prolonged complete atrioventricular block (CAVB), were investigated. The frequency of paroxysmal Af in response to ACh provocation was also examined. In the case of the occurrence of Af, the involved vessel, the provocation dose administered, and the need for further drug treatment were recorded.

### 2.4. Parameters

Data on family history of coronary artery disease (CAD), current smoking habits, and alcohol consumption were gathered. Hypertension was defined using conventional methods [16]. Using the accepted formula [19], the estimated glomerular filtration rate (eGFR) (mL/min/1.73 m^2^) was determined, and the existence of chronic kidney disease (CKD) was determined according to accepted standards. The use of drugs for dyslipidemia and a low-density lipoprotein cholesterol level of ≥120 mg/dL were both deemed as evidence of dyslipidemia. Hemoglobin (Hb) A1c levels of ≥6.5%, a fasting blood sugar level of ≥126 mg/dL, and the use of anti-diabetes mellitus (DM) drugs were defined as evidence of DM. Echocardiography was used to calculate the left ventricular ejection fraction (LVEF). The types of drugs taken at admission were checked. Regarding the number of coronary vasodilators, as a rule, coronary dilators were stopped 48 h before the SPT; however, we examined the number that had been taken before discontinuation. Cardiac medical history, involving elements such as a history of percutaneous coronary intervention (PCI), previous myocardial infarction, and heart failure, was also assessed. The frequencies of the aforementioned parameters and VSA-related factors were compared between the RCA and LCA groups.

### 2.5. Statistical Analyses

Continuous variables with normal distributions are expressed as means and standard deviations whereas continuous variables with non-normal distributions are expressed as medians (interquartile ranges). Categorical variables are expressed as frequencies (%). Student’s unpaired t-test, the Wilcoxon signed-rank test, or the chi-square test was used to compare the baseline characteristics between the two groups.

The time period of this study was different, leading to differences in the dosage and type of provocative drugs during the SPT; therefore, we performed propensity score matching (PSM) analyses as sensitivity analyses. We estimated the propensity score for the RCA or LCA group using a logistic regression model that included all baseline variables as covariates. Then, we conducted 1:1 matching of patients between the RCA and LCA groups with the closest estimated propensity score within a caliper (≤0.20 of the pooled standard deviation of estimated logits) using the nearest neighbor method without replacement.

All statistical analyses were performed using JMP (version 17; SAS Institute Inc., Cary, NC, USA). *p*-values < 0.05 were used to denote statistical significance.

## 3. Results

### 3.1. Patient Characteristics

Of the 225 patients enrolled in this study, 133 (59%) were assigned to the RCA group and 92 (41%) were assigned to the LCA group. No cases of SPT were discontinued during the study.

The patient characteristics are shown in Table 1. No significant differences in age, sex, frequency of traditional cardiovascular risk factors, and other data were observed between the two groups. Regarding the drugs taken upon admission, the LCA group used statins more frequently than the RCA group (*p* = 0.029); the frequency and numbers of coronary vasodilators were not significantly different between the two groups.

Using 1:1 PMS, 60 pairs were generated (Figure 1). After PMS, the prevalence of all variables was similar in both groups (Table 1).

### 3.2. CAG and SPT Results

**Figure 2:**
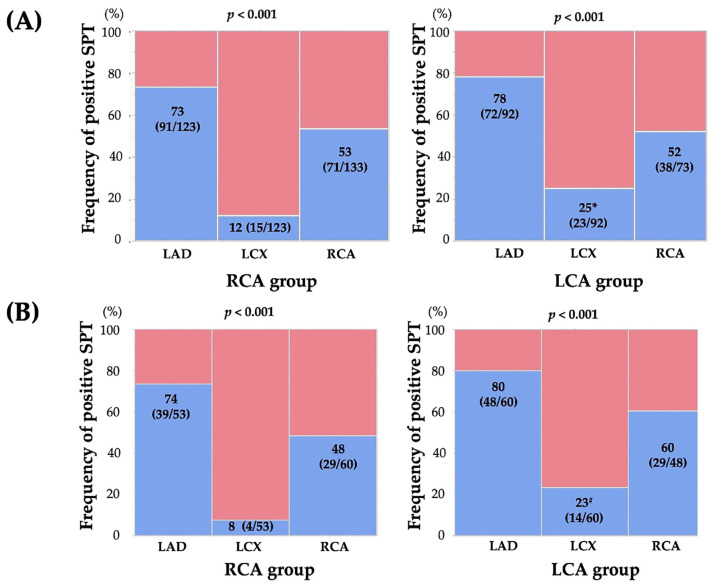
Frequencies of positive SPT in each coronary artery in the RCA and LCA groups for all patients (**A**) and propensity score-matched patients (**B**). The symbols * and ^#^ indicate significant differences (*p* = 0.015 and *p* = 0.022, respectively) in the frequency of positive coronary spasms in the LCX in the LCA group compared with that in the RCA group. Abbreviations: LAD, left anterior descending coronary artery; LCA, left coronary artery; LCX, left circumflex coronary artery; RCA, right coronary artery.

The results of the SPT and CAG are summarized in Table 2. The frequency of atherosclerotic lesions tended to be lower in the LCA group than in the RCA group whereas no significant difference in the frequency of significant coronary stenosis was observed between the two groups. Regarding the SPT, the doses of ACh were higher in the LCA group than in the RCA group (*p* < 0.001), and the use of EM and sequential provocation showed higher trends in the LCA group than in the RCA group. No significant difference in the SPT positivity rate was observed between the two groups (79% in the RCA group and 85% in the LCA group; *p* = 0.269). Moreover, no significant differences in the frequencies of the unavoidable use of NTG and focal spasms were observed between the two groups. Regarding the location of the spasm-provoked coronary artery, the frequency of coronary spasms in the LCX was higher in the LCA group (25%) than in the RCA group (12%) (*p* = 0.016) whereas no significant difference in the frequencies of coronary spasms in the LAD and RCA was observed between the two groups (Figure 2). This finding was the same in patients who underwent the SPT without high doses of ACh, EM, or sequential provocation (14% [13/94] in the RCA group, 30% [15/50] in the LCA group; *p* = 0.020) and in patients without the unavoidable use of NTG in the RCA group (12% [12/101] in the RCA group, 25% [23/92] in the LCA group; *p* = 0.018). In each group, the coronary artery tree in which coronary spasms were most frequently provoked was the LAD (*p* < 0.001) (Figure 2).

After PSM, no significant difference in the SPT positivity rate was observed between the two groups (82% in the RCA group and 88% in the LCA group; *p* = 0.307). Regarding the location of the spasm-provoked coronary artery, the frequency of coronary spasms in the LCX remained higher in the LCA group (23% [14/60]) than in the RCA group (8% [4/53]) (*p* = 0.022). Except for high doses of ACh, EM, or sequential provocation, the frequency of coronary spasms in the LCX remained higher in the LCA group (28% [10/36]) than in the RCA group (6% [2/33]) (*p* = 0.017). In each group, the coronary artery tree in which coronary spasms were most frequently provoked was still the LAD (*p* < 0.001) (Figure 2).

Regarding the assessment of MVS, because of the presence of patients with ND, we could assess MVS in 123 patients in the RCA group and 78 patients in the LCA group. No significant difference in the frequency of MVS was observed between the two groups (50% in the RCA group and 62% in the LCA group; *p* = 0.122). A significant difference in the locations of coronary arteries with MVS was observed between the two groups (*p* < 0.001). The MVS pattern in the RCA group was more frequently observed in the “LAD and RCA (79%)”, whereas that in the LCA group was more frequently observed in the “LAD and RCA” and “LAD and LCX” (*p* < 0.001). The pattern of MVS differed between the two groups (*p* < 0.001). The MVS pattern in the RCA group was most common in the “LAD and RCA” (79%), while the “LAD and LCX” was absent (0%). In contrast, that of the LCA group was similar, with the “LAD and RCA” being the most common (54%) but being observed second to the “LAD and LCX” (27%).

After PSM, the frequency of MVS was higher in the LCA group (62%) than in the RCA group (40%) (*p* = 0.020). A significant difference in the MVS pattern was observed between the two groups (*p* = 0.024). The MVS pattern in the RCA group was most common in the “LAD and RCA” (90%), while the “LAD and LCX” was none (0%). In contrast, that of the LCA group was similar, with the “LAD and RCA” being the most common (54%) but being observed second to the “LAD and LCX” (21%).

### 3.3. SPT-Related Complications

The frequencies of SPT-related complications are shown in Table 2. Af occurred in thirty-eight (17%) cases overall, with twenty-five (66%) in the RCA, ten (26%) in the LCA, and three (8%) in both the RCA and LCA, being more common in the RCA. No significant difference in the frequency of paroxysmal Af was observed between the two groups (17% in the RCA group and 16% in the LCA group; *p* = 0.846). A significant difference in the timing of Af was found between the two groups (*p* = 0.018). In the RCA group, Af occurred when the SPT was performed in the RCA (*n* = 19), in the LCA (*n* = 3), and in both the RCA and LCA (*n* = 1). In the LCA group, Af occurred when the SPT was performed in the LCA (*n* = 7), in the RCA (*n* = 6), and in both the LCA and RCA (*n* = 2). Af occurred after the administration of high doses of ACh in 26% (6/23) of the patients in the RCA group and in 40% (6/15) of the patients in the LCA group. The rate of the use of antiarrhythmic drugs, such as cibenzoline, was 52% (12/23) in the RCA group and 80% (12/15) in the LCA group (*p* = 0.082).

No significant difference in the incidence of severe complications such as Vf or pVT, cardiac arrest or prolonged CAVB, or unstable hemodynamics necessitating catecholamine infusion was observed between the two groups (3% in the RCA group, 5% in the LCA group) (*p* = 0.361) One case of cardiac arrest was complicated by CABV and pacemaker malfunction when ACh was loaded into the RCA, resulting in P waves only, and the patient recovered just before chest compressions were started.

After PSM, the frequency of SPT-related complications, including Af, was comparable between the two groups (Table 2). No significant differences in the frequencies of paroxysmal Af (12% in the RCA group and 13% in the LCA group; *p* = 0.783) and severe complications (3% in the RCA group and 5% in the LCA group; *p* = 0.648) were observed between the two groups.

## 4. Discussion

In this retrospective observational study, we examined the differences in the frequency of positive SPT (including MVS) and SPT-related complications between patients who underwent SPT starting from the RCA or LCA. The frequencies of positive SPT (including MVS) and complications, including Af, were comparable between the two groups. Furthermore, we found that LAD was most likely to be positive regardless of the choice of whether the RCA or LCA was used to start the SPT. Furthermore, the LCX was more likely to be positive if the SPT was initiated from the LCA. Furthermore, after PSM, we demonstrated that the LCX was still more likely to be positive if the SPT was initiated from the LCA, leading to a higher frequency of MVS in the LCA group than in the RCA group. Thus, although the choice of using the RCA or LCA for starting the SPT should be considered based on the patients’ symptoms and CAG findings, a routine SPT may be started from the LCA.

The SPT is an indispensable test in diagnosing and treating VSA because it provides information on the activity of VSA [6]. Its high diagnostic rate and good safety profile make it an excellent test; nevertheless, the question of which coronary artery to perform the SPT from has been addressed by several studies [6]. MVS is also an important prognostic indicator [8], and it is recommended that if the SPT is to be performed, a diagnosis of MVS should be made whenever possible [6]. At our hospital, the SPT was performed mainly from the RCA until April 2016 and mainly from the LCA after that. Thus, in this study, we compared the results for approximately 2 years before and after that time. We observed no difference in the diagnostic rate of VSA, regardless of whether the SPT was initiated from the RCA or LCA in all patients. However, the study population included many patients with a high probability of VSA, and because of the changes in the doses of ACh and/or in the SPT methods during the observed periods, our results should be carefully interpreted. Sueda and Saito et al. reported on the changed dosage of ACh [12,18,20]. In particular, Sueda et al. reported that the maximum ACh dosage and sequential provocation [12,18] may increase the diagnostic yield, and we have adopted these methods. High-dose ACh provocation was used more frequently in the LCA group, which may have influenced the results of this study. To lessen such effects on the results, the analyses were performed using PSM, the results of which showed no difference in the frequency of VSA but a significantly higher frequency of MVS in the LCA group. Considering these results, the SPT may start from the LCA to raise the diagnostic capacity of MVS; nevertheless, the results of this study should be confirmed in a multicenter registry that includes several patients or a prospective study with uniform provocative doses.

Generally, it has been shown that SPT positivity in the LCX is lower than in the other two coronary arteries, and the difference in muscarinic receptor distribution in coronary arteries may account for this finding [21]. Even in this study, the low frequency of SPT positivity in the LCX was confirmed. However, in this study, we demonstrated that SPT positivity in the LCX was significantly higher in the group in which the SPT was initiated from the LCA than in the group where the SPT was initiated from the RCA. Even though cases with high-dose ACh provocation or those in which provocation was started from the RCA and NTG was administered to the RCA were excluded from the analyses, provocation from the LCA resulted in a high frequency of LCX positivity compared with provocation from the RCA. Furthermore, this finding was confirmed by the analyses after PSM. These findings suggest that the LCX becomes less prone to coronary spasms as it is exposed to ACh provocation and becomes accustomed to the stimulus. This hypothesis should be clarified using multicenter prospective studies. In any case, if the involvement of the LCX in coronary spasms is to be clarified, the SPT may be started from the LCA. Furthermore, the frequency of coronary spasms in the LAD and RCA also varied between reports [21,22,23]; Sueda et al. [21] reported no difference in the positivity rate between the RCA (73.3%) and LAD (72%). A large study using ergonovine stress echocardiography found 43% of wall motion abnormalities to be in the LAD region [22]. Jin et al. reported a higher frequency of positive coronary spasms for the LAD (64.3%) in probable positive definition and the RCA (65.4%) in positive definition, albeit in a few cases [23]. In our study, the frequency of coronary spasms in the LAD was clearly higher. It is possible that our results may have been the way they were because of the inclusion of patients who were quite likely to have VSA and the differences in provocative agents [24]. Future studies are required to determine whether the frequency of coronary spasms in the LAD is also higher.

Considering SPT-related complications, 20% (45/225) of all patients had these complications, of whom 4% (9/225) had severe complications and 17% (38/225) had paroxysmal Af. It has been shown that SPT-related complications are more common with ACh than with EM [25,26] and that they are more likely to occur in patients from Oriental regions than in those from Western regions [26,27]. Although body size and the time of administration of the drug may have an effect, our results seemed to be more frequent than those of other studies in Japan [25,26]. Although the administration times and intervals are standardized within our facility, they may differ across attending physicians due to the manual administration of provocative drugs. Carefully performing SPT is necessary in the future. The rate of paroxysmal Af has been reported to be 8–17% [28,29], which may be influenced by whether the LCA or RCA is being provocative and by what provocation dose is being applied. Our data may have a higher frequency of Af; however, they do not seem to deviate from the conventional direction. One of the concerns about performing the SPT from the RCA is the possibility of complications including arrhythmias. However, in this study, where all patients underwent temporary pacing during the SPT, no significant difference in the occurrence of complications, including arrhythmias, was observed between the two groups. Thus, it is safe to conclude that the SPT can be performed from either the LCA or RCA. Saito et al. reported that Af is more likely to occur when the SPT is started from the RCA [29]. In this study, Af also occurred more frequently during RCA provocation in the group wherein the SPT was initiated from the RCA. In contrast, in the LCA group, no significant difference in the frequency of Af was observed between LCA and RCA provocations. Although Af may be more likely to occur during RCA provocations, which is consistent with the coronary spasm-positive mechanism of the LCX described above, we hypothesized that Af is more likely to occur during the first ACh-loaded stimulation. In any case, clarifying which coronary artery is more likely to produce Af when the SPT is initiated and whether Af produced during RCA provocation is more likely to resolve spontaneously is necessary.

The clinical implications of this study are as follows. The coronary artery from which the SPT is initiated should be determined based on the patients’ medical condition, institutional expertise and familiarity with the SPT, and angiographic findings. If the vessel diameter of the RCA is relatively small, the SPT may often be started with the LCA. In addition, if the SPT is to be performed under close examination for syncope, it should be preferably started in the RCA because of the possibility of coronary spasms in the RCA [30]. Catheter-induced coronary spasms are also considered actual instances of VSA in many cases; therefore, the SPT may be performed on the RCA if such a finding exists [31]. Furthermore, focal spasms tend to occur in atherosclerotic lesions [32,33] and are a factor that influences the prognosis [7,10]; therefore, if an atherosclerotic lesion is present, the SPT may be performed on that vessel first. However, considering our results, including PSM analyses, which showed that coronary spasms occur more frequently in the LAD and more likely in the LCX if the SPT is started from the LCA, a routine SPT may be started from the LCA. Furthermore, if time is limited for any examination or if the use of contrast media is limited due to decreased renal function, cardiologists may start the SPT with the LCA because MVS can be evaluated only with the LCA. Furthermore, the difference in prognosis between the three-vessel spasm (3-VS) and two-vessel spasm (2-VS) is unclear [15]; however, if no difference in prognosis is found between patients with VSA with between 3-VS and 2-VS, 2-VS should be evaluated first, and even then, performing the SPT from the LCA may be beneficial for skipping RCA spasms if coronary spasms occur in the LAD and LCX.

While interpreting the findings of this study, certain limitations should be considered. First, because this was a single-center retrospective study, selection bias might have been a factor. Furthermore, the number of patients under study was small. Second, as mentioned above, the ACh dosage was different and more aggressively provocative in the LCA group. Furthermore, the frequencies of dyslipidemia were similar in the two groups; however, the frequency of statin use was significantly higher in the LCA group. Because statins are also therapeutic agents in VSA [5,34,35], the frequency of statin use may have increased over time. Therefore, some analyses were performed using PSM to reduce these effects; however, PSM may not always be sufficient because of the small number of patients under study after PSM. Third, the choice of ACh provocation dose and treatment of Af were at the discretion of the attending physician, and their frequencies may vary depending on the attending physician’s decision even within the same facility. Fourth, an ECG performed during chest symptoms provides essential information, particularly when showing ST-segment elevation, which is considered a prognostic factor [8,36]. However, we do not routinely perform Holter ECGs at our institution; therefore, we were unable to present this information in this study. Finally, other imaging studies and tests for coronary microcirculation indices are often performed along with the SPT [33], and contrast media use and the overall procedure time are affected by these tests; however, we are unable to present these data at this time.

## 5. Conclusions

In this study, no significant differences in the frequencies of positive SPT and SPT-related complications were observed between patients who underwent the SPT starting from the RCA and those who underwent the SPT starting from the LCA; however, after PSM, the frequency of MVS, due to the increase in coronary spasms in the LCX, was higher when the SPT was initiated from the LCA. The choice of the coronary artery to be used for the SPT should be based on the patient’s symptoms, institutional expertise and familiarity with the SPT, and coronary angiographic findings; however, a routine SPT may be started from the LCA. Future studies should clarify whether an SPT initiated from the LCA or RCA is more likely to produce coronary spasms in the LCX.

## Figures and Tables

**Table 1:** Characteristics of the study population.

		All Patients (*n* = 225)			Propensity Score-Matched Patients (*n* = 120)		
		RCA Group	LCA Group	*p*-Value	RCA Group	LCA Group	*p*-Value
*n* (%)		133 (59)	92 (41)		60 (50)	60 (50)	
Men/Women		63/70	53/39	0.461	31/29	30/30	0.855
Age (years)		66 ± 12	67 ± 13	0.577	66 ± 11	66 ± 13	0.965
Body mass index (kg/m^2^)		24.4 ± 4.1	24.0 ± 4.0	0.481	23.9 ± 3.7	23.9 ± 3.8	0.969
Family history of CAD		28 (21)	15 (16)	0.373	9 (15)	10 (17)	0.803
Alcoholic drinker (%)		41 (31)	37 (40)	0.146	24 (40)	24 (40)	1.00
Cardiovascular risk factors (%)							
	Current/past/never smoker	22/37/73	16/23/53	0.881	10/17/33	11/18/32	0.978
	Hypertension	83 (63)	62 (67)	0.487	39 (65)	39 (65)	1.000
	Dyslipidemia	74 (56)	51 (55)	0.926	37 (62)	33 (55)	0.459
	Diabetes mellitus	24 (18)	17 (18)	0.955	12 (20)	13 (22)	0.822
eGFR (mL/min/1.73 m^2^)		70.6 ± 16.2	67.6 ± 15.3	0.142	70.7 ± 16.0	69.9 ± 15.5	0.747
Presence of CKD (%)		37 (28)	27 (29)	0.830	14 (23)	15 (25)	0.831
LVEF on echocardiography (%)		67 ± 8	67 ± 7	0.850	68 ± 7	67 ± 7	0.687
BNP (pg/mL)		21 (12, 43)	20 (9, 40)	0.341	22 (10, 51)	20 (10, 39)	0.532
Cardiac complications							
	Post PCI (%)	5 (4)	4 (4)	0.825	4 (7)	4 (7)	1.000
	Previous myocardial infarction (%)	1 (1)	3 (3)	0.162	1 (2)	1 (2)	1.000
	Heart failure (%)	2 (2)	1 (1)	0.789	1 (2)	1 (2)	1.000
Medications							
	Calcium channel blockers (%)	48 (36)	32 (35)	0.840	23 (38)	21(35)	0.705
	Long-acting nitrates (%)	13 (9)	8 (9)	0.785	2 (3)	4 (7)	0.402
	Nicorandil (%)	6 (5)	9 (10)	0.119	4 (7)	4 (7)	1.000
	No. of coronary vasodilators	0.5 ± 0.6	0.5 ± 0.8	0.762	0.5 ± 0.7	0.5 ± 0.6	1.000
	RAS inhibitor (%)	31 (23)	23 (25)	0.777	12 (20)	12 (20)	1.000
	Diuretics (%)	9 (7)	3 (3)	0.250	3 (5)	3 (5)	1.000
	β blockers (%)	13 (10)	11 (12)	0.602	7 (12)	7 (12)	1.000
	Statin (%)	43 (32)	43 (47)	0.029	28 (47)	24 (40)	0.461
	Anti-platelet therapy (%)	32 (24)	23 (25)	0.872	17 (28)	19 (32)	0.690
	Oral diabetes medication (%)	15 (11)	11 (12)	0.876	8 (13)	7 (12)	0.783
	Insulin (%)	1 (1)	2 (2)	0.361	0 (0)	0 (0)	(−)

Categorical variables are expressed as frequencies (percentages) and continuous variables are expressed as means ± standard deviations or medians (interquartile ranges). Abbreviations: BNP, brain natriuretic peptide; CAD, coronary artery disease; CKD, chronic kidney disease; eGFR, estimated glomerular filtration rate; LCA, left coronary artery; LVEF, left ventricular ejection fraction; PCI, percutaneous coronary intervention; RAS, renin–angiotensin system; RCA, right coronary artery.

**Table 2:** Results of the SPT and CAG.

				All Patients (*n* = 225)			Propensity Score-Matched Patients (*n* = 120)		
				RCA Group	LCA Group	*p*-Value	RCA Group	LCA Group	*p*-Value
*n* (%)				133 (59)	92 (41)		60 (50)	60 (50)	
CAG									
	Atherosclerotic lesions (%)			67 (50)	35 (38)	0.068	26 (43)	30 (50)	0.464
SPT									
	Use of high doses ACh (%)			30 (23)	42 (46)	<0.001	20 (33)	24 (40)	0.449
	Use of EM (%)			4 (3)	8 (9)	0.062	3 (5)	3 (5)	1.000
	Use of sequential provocation (%)			1 (2)	4 (4)	0.072	1 (2)	1 (2)	1.000
	Positive SPT (%)			105 (79)	78 (85)	0.269	49 (82)	53 (88)	0.307
	Use of unavoidable NTG (%)			32 (24)	27 (29)	0.375	15 (25)	17 (28)	0.680
	Presence of focal spasm (%)			37 (28)	33 (36)	0.200	19 (32)	27 (45)	0.133
	Location of spasm-provoked coronary artery								
		LAD spasm (*n*, %)		91 (123, 73)	72 (92, 78)	0.410	39 (53, 74)	48 (60, 80)	0.419
		LCX spasm (*n*, %)		15 (123, 12)	23 (92, 25)	0.015	4 (53, 8)	14 (60, 23)	0.022
		RCA spasm (*n*, %)		71 (133, 53)	38 (73, 52)	0.855	29 (60, 48)	29 (48, 60)	0.211
	MVS (*n*, %)			62 (123, 50)	48 (78, 62)	0.122	21 (53, 40)	33 (53, 62)	0.020
		LAD + RCA/LAD + LCX/LCX + RCA/3-VS		49/0/2/11	26/13/0/9	<0.001	19/0/0/2	19/7/0/7	0.024
	Complications			25 (19)	20 (22)	0.588	9 (15)	11 (18)	0.624
		Paroxysmal Af (%)		23 (17)	15 (16)	0.846	7 (12)	8 (13)	0.783
		Severe complications (%)		4 (3)	5 (5)	0.361	2 (3)	3 (5)	0.648
			Vf or pVT (%)	1 (1)	3 (3)	0.162	1 (2)	1 (2)	1.000
			Cardiac arrest or prolonged CAVB (%)	1 (1)	1 (1)	0.792	0 (0)	1 (2)	0.315
			Use of catecholamine infusion (%)	3 (2)	1 (1)	0.514	1 (2)	1 (2)	1.000

Data are expressed as frequencies (percentages or numbers in some data). Abbreviations: ACh, acetylcholine; Af, atrial fibrillation; CAG, coronary angiography; CAVB, complete AV block; EM, methylergometrine maleate; LAD, left anterior descending coronary artery; LCA, left coronary artery; LCX, left circumflex coronary artery; MVS, multivessel spasm; NTG, nitroglycerin; pVT, pulseless ventricular tachycardia; RCA, right coronary artery; SPT, spasm provocation test; Vf, ventricular fibrillation; VS, vessel spasm.

## Data Availability

Not applicable.
